# Cognitive processing of cluster headache patients: evidence from event-related potentials

**DOI:** 10.1186/1129-2377-15-66

**Published:** 2014-10-02

**Authors:** Rongfei Wang, Zhao Dong, Xiaoyan Chen, Ruozhuo Liu, Mingjie Zhang, Jinglong Wu, Shengyuan Yu

**Affiliations:** 1 Department of Neurology, Chinese PLA General Hospital, Fuxing Road 28, Haidian District, Beijing 100853, China; 2 Medical school, Nankai University, Tianjin, China; 3 Biomedical Engineering Laboratory, Graduate School of Natural Science and Technology, Okayama University, 3-1-1 Tsushima-Naka, Okayama 700-8530, Japan

**Keywords:** Cluster headache, ERPs, P3/P3d, Cognitive processing

## Abstract

**Background:**

The peripheral and central origins of pain in cluster headache (CH) have been a matter of much debate. The development and application of functional imaging techniques have provided more evidence supporting the hypothesis that CH is not a disorder exclusively peripheral in origin, and in fact central regions might be more important. Event-related potentials confer advantages in the functional evaluation of the cortex, but few studies thus far have employed this method in cluster headache.

**Methods:**

Seventeen cluster patients (15 males; mean age = 35.4 years) and 15 age-matched healthy participants (13 males; mean age = 34.6 years) were recruited. A visual oddball paradigm was employed to analyze target processing using event-related potentials. We investigated the P3/P3d components in the experiment.

**Results:**

P3/P3d amplitudes were decreased in CH patients (P3, 3.82 μV; P3d, 5.8 μV) compared with controls (P3, 7.28 μV; P3d, 8.95 μV), *F*(1,30) = 4.919, *p* < 0.05, η2 = 0.141 for P3 and *F*(1,30) = 8.514, *p* < 0.05, η2 = 0.221 for P3d, respectively). Moreover, the amplitudes of P3/P3d were no significantl difference in the side of pain as compared to contralateral one (p > 0.05).

**Conclusions:**

These results provide evidence of dysfunction in the cognitive processing of CH patients, which may also contribute to the pathophysiology of CH.

## Background

Cluster headache (CH) causes severe unilateral temporal or periorbital pain, usually lasting between 15 and 180 minutes, and is accompanied by autonomic symptoms in the nose, eyes, and face. Headaches often recur at the same time each day during the cluster period, which can last for weeks or even months. CH is more prevalent in men, and its typical onset is 20–40 years of age. A Chinese clinic-based study, approximately 1 year in duration, estimated that trigeminal autonomic cephalalgia accounts for 5.3% of primary headaches, of which 84.7% are CH [1]. However, the pathophysiology of CH is not yet understood fully.

Central structures play an important role in the etiology of CH. The development of functional imaging techniques has been invaluable for the development of theories pertaining to the central mechanisms of CH. Several functional imaging studies have demonstrated the involvement of the hypothalamus [2-4]; in addition, activation in certain areas of the pain neuromatrix, which includes the thalamus, anterior cingulate cortex (ACC), insula, basal ganglia, cingulum, frontal cortex, and the cerebellar hemispheres, has also been reported during the acute pain state [4-7]. Cognitive processing studies employing event-related potentials (ERPs) support the hypothesis that CH cannot be exclusively peripheral in origin [8-11]. Many researchers report that brain structures involved in cognitive processing also underlie the pathophysiology of CH [8].

ERPs reveal coherent stimulus-related postsynaptic activity in the cortices, with a temporal resolution measurable in milliseconds. Accordingly, ERPs are ideally suited for investigation of the cortical activation time course during cognitive processing and also confer advantages during functional cortical evaluation. Several ERP studies have been conducted in CH patients in an effort to understand certain higher brain abnormalities. For example, Evers et al. [9,10] reported an increase in P3 latency in CH patients during the cluster period in a visual ERP test. Because P3 latency is an indicator of cognitive performance [11], the authors concluded that cognitive processing is impaired during the cluster period, lending credence to the notion that CH has a central origin. However, other neuropsychological tasks did not reveal significant abnormalities in CH patients (pertaining to their cognitive processing) [12]. Furthermore, personality studies have implied that CH patients do not experience learning disabilities [13,14]. The above studies also failed to demonstrate changes in the amplitude of P3, mainly because of the type of patients recruited and the difficulty involved in effecting stimulations.

In the present study, we used a traditional visual oddball paradigm, in which participants were required to press a button for the infrequent target stimulus, while ignoring the frequent non-target standard stimulus. We focused on P3 (a generic name for a variety of relatively late positive components [15,16]).

## Methods

### Participants

We recruited 17 cluster patients (15 males; mean age, 35.4 years; range, 20–45 years) from the Chinese PLA General Hospital according to the diagnostic criteria of the International Classification of Headache Disorders (3rd ed., beta version; ICHD-3 beta). The CH duration among patients ranged from 5 to 15 years, and the cluster period ranged from 1 to 6 months. The frequency of CH attacks during previous cluster periods was 1–3 attacks per day. Patients were not receiving prophylactic therapy, were drug-free for at least 24 hours, and were in bout but not in headache when recruited. We also recruited 15 healthy age-matched participants (13 males; mean age, 34.6 years; range, 22–43 years) with no history of headache or drug/alcohol abuse. Patients and controls had normal or corrected-to-normal vision and normal hearing capabilities. No participants had notable motor or sensory dysfunction or deep tendon reflexes. We excluded participants who were illiterate or suffering from depression, stroke, or brain injuries. The study was approved by the Ethical Committee of the Chinese PLA General Hospital in accordance with the ethical principles of the Declaration of Helsinki. All participants provided written and informed consent prior to commencement of the experiment.

The following clinical data of the CH patients were included: 1) past history of CH; 2) frequency of headaches over the previous year; 3) ratings for the most severe headache experienced during the previous year using a visual analog scale (VAS); and 4) position of the headache. The exclusion criteria were as follows: 1) taking prophylactic medications for CH; 2) history of analgesic drug overuse; 3) history of general neurological or psychiatric disease; 4) history of drug abuse or dependency, including alcohol and cigarettes; 5) history of mixed-type headache; and 6) past history of neurological disorder or abnormal findings on a neurological examination.

The evaluation of the Montreal Cognitive Assessment (MoCa) and Mini-Mental Status Examination (MMSE) scores were carried out in all the participants.

### Stimuli and procedures

**Figure 1:**
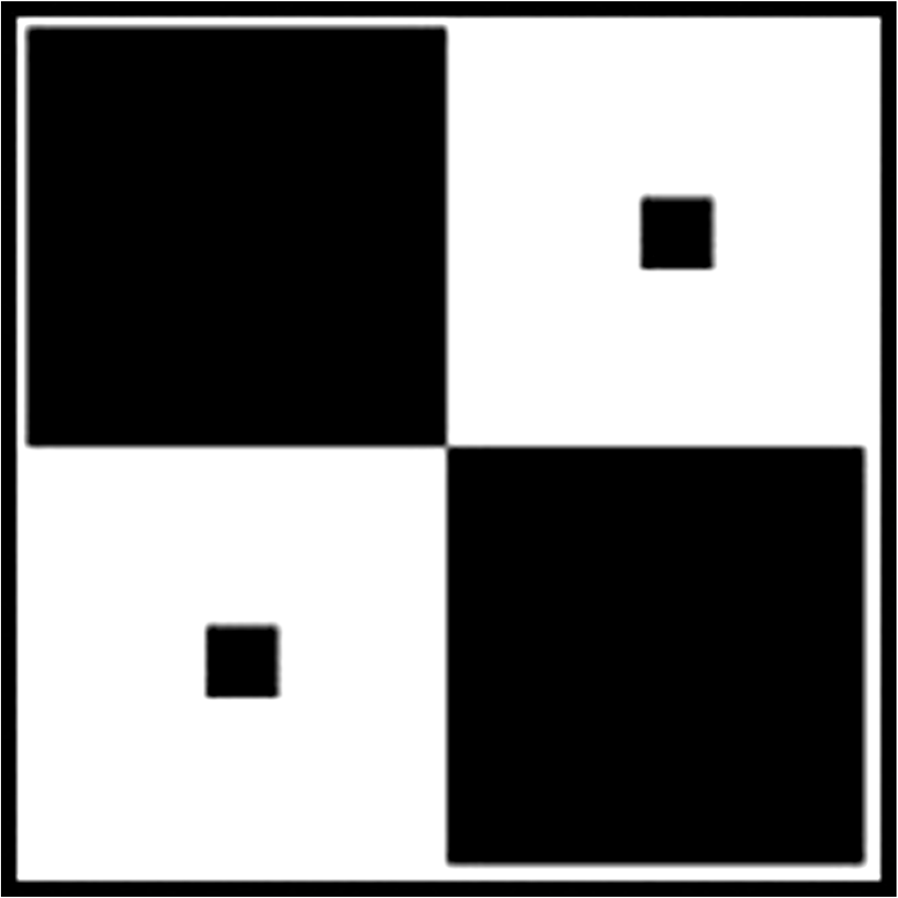
The picture showed the appearance of the target stimulus.

**Figure 2:**
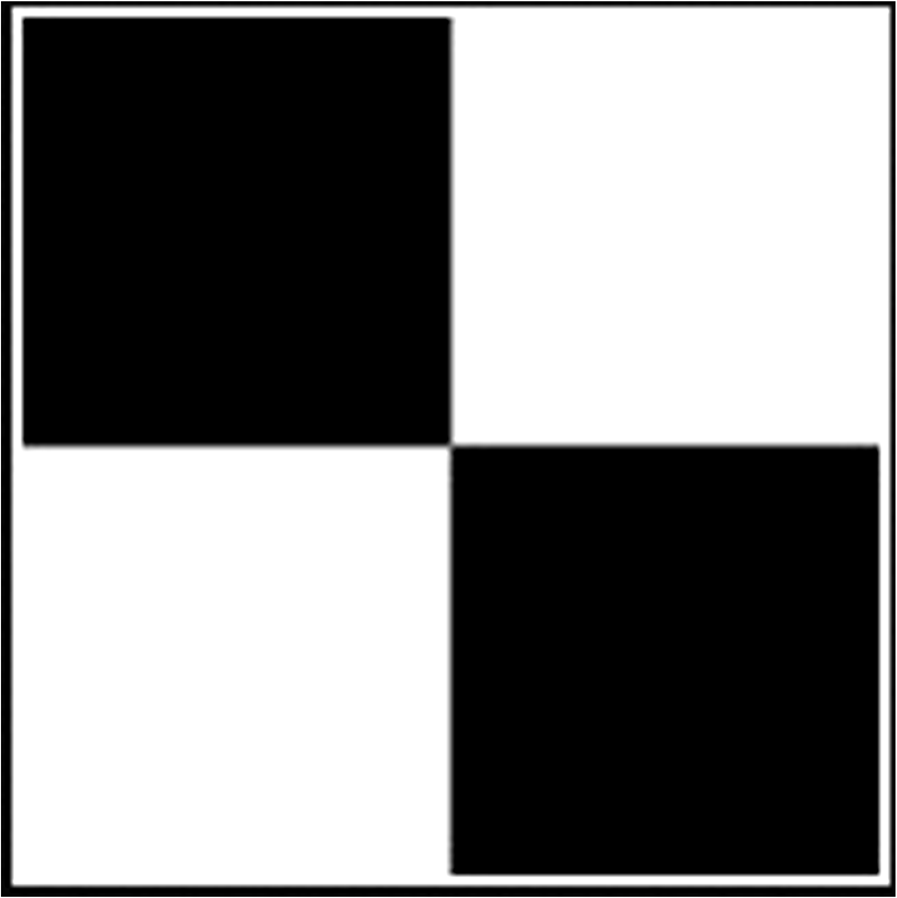
The picture showed the appearance of the standard stimulus.

The experiment was performed in a sound-attenuated room with a dim light. Stimuli comprised target stimuli (Figure 1, 20% probability of presentation) and standard stimuli (Figure 2, 80% probability of presentation). The duration of both stimuli was 105 ms. The inter-stimulus interval (ISI) varied randomly between 1,000 and 1,500 ms (mean = 1,200 ms). Two separate blocks, each comprising 160 stimuli, were presented. All the stimuli were showed out by the E-prime software.Participants were instructed to focus on a fixation cross in the center of the screen and to press the button as quickly and accurately as possible when they viewed the target stimuli (Figure 1). The accuracy was the rate of pressing the button when viewed the target stimuli. The appropriate reaction time was the length between viewing the target stimuli and pressing the button accurately. The accuracy and appropriate reaction time were recorded by the E-prime software.

### EEG recording and analysis

An electroencephalogram (EEG) was recorded continuously (band pass 0.05-100 Hz, sampling rate 500 Hz) at the F3, F4, Fz, C3, C4, Cz, P3, P4, and Pz electrode sites, according to the international 10–20 system and using an ASA-Lab EEG/ERP 64-channel amplifier (http://www.ant-neuro.com) referenced to the left mastoid (right mastoid was used as the recording site). vertical electroculogram (VEOG) and horizontal electroculogram (HEOG) were recorded using two pairs of electrodes, one pair placed above and below the right eye, and the other placed 10 mm from the lateral canthi. Electrode impedance was maintained below 5 kΩ throughout the experiment.

**Figure 3:**
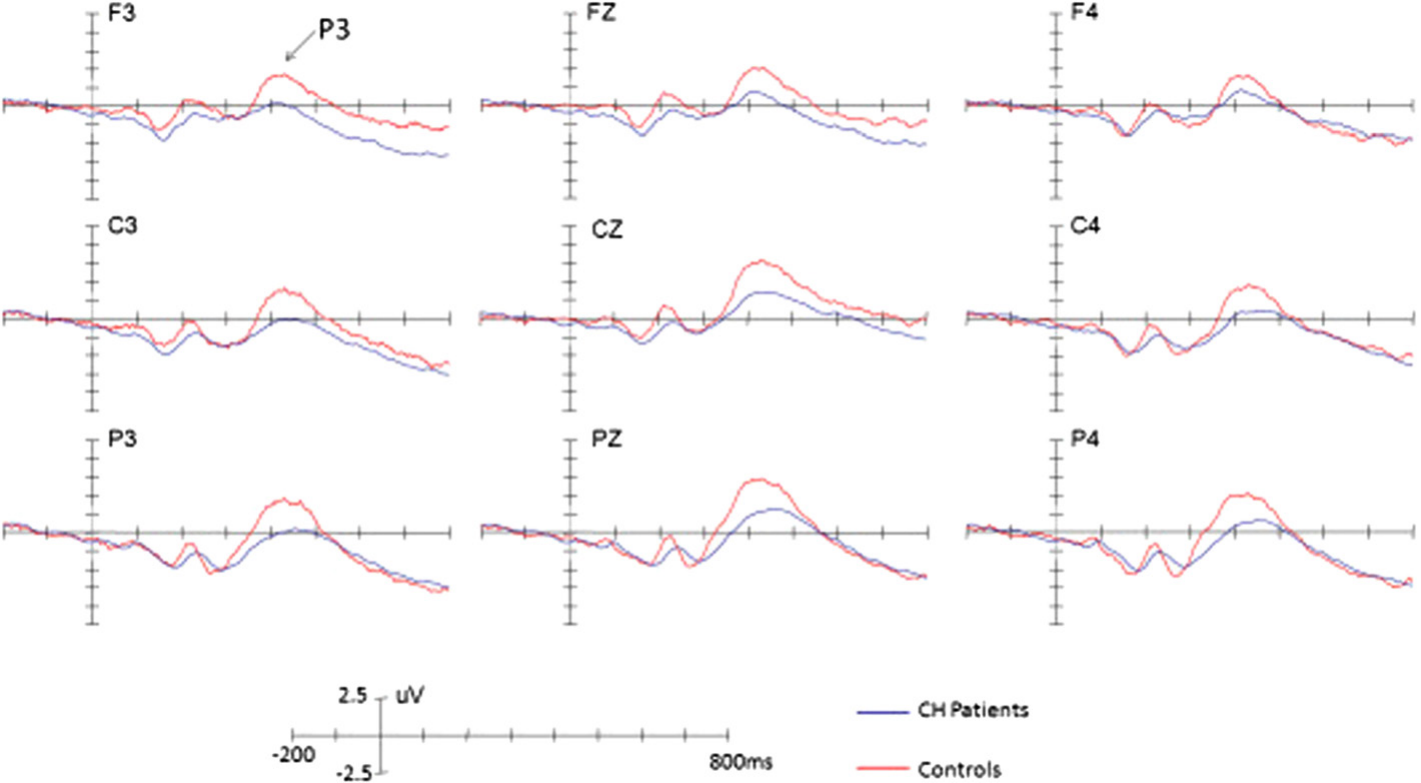
The grand averaged ERPs elicited by target stimuli in patients and controls, respectively.

**Figure 4:**
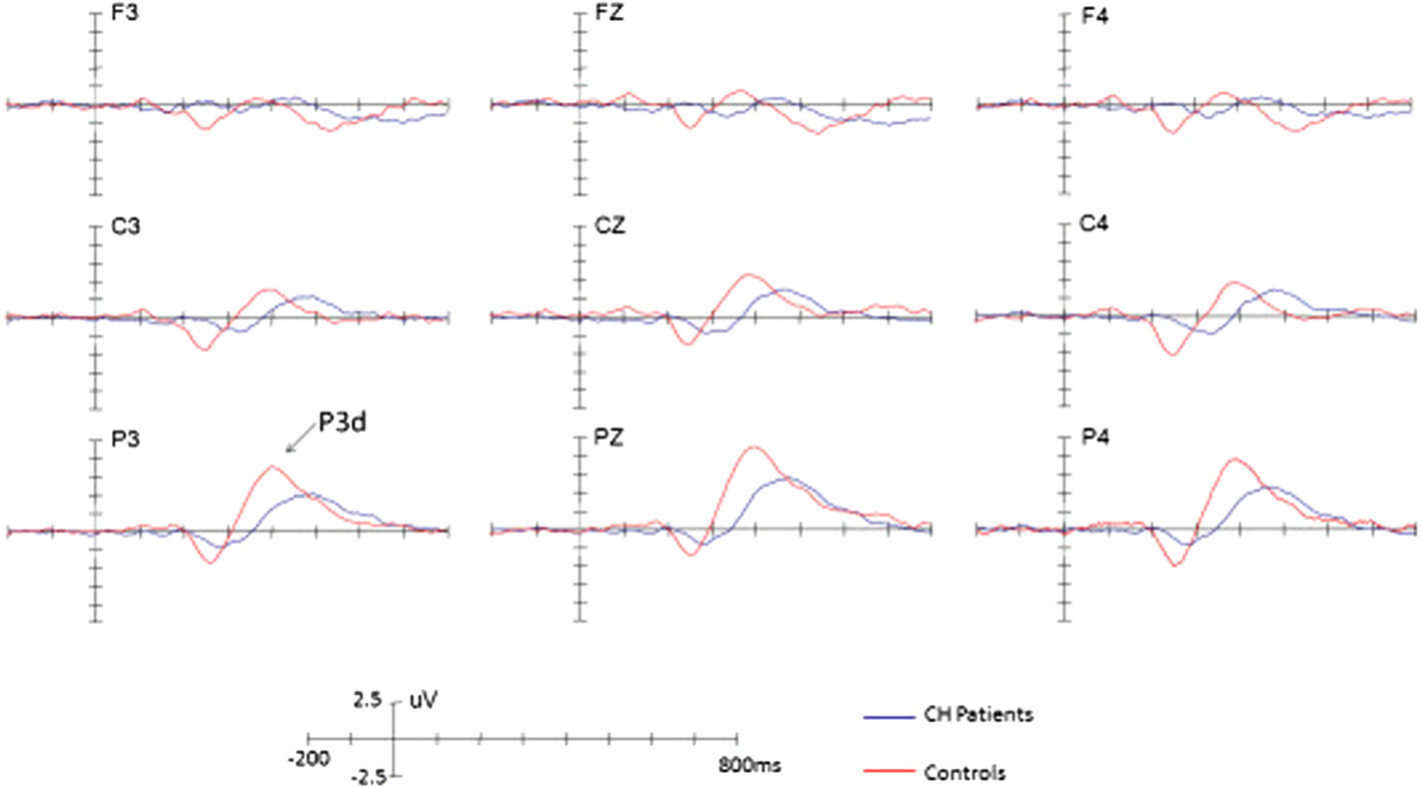
The difference waveforms by subtracting ERPs in response to standard stimuli from ERPs in response to target stimuli in patients and controls, respectively.

We used ASA software (http://www.ant-neuro.com) to analyze the data offline. EEG data were re-referenced to the bi-mastoid average reference. EOG artifacts were corrected using the method proposed by Semlitsch et al. (1986). EEG was segmented into the epoch running from 200 ms pre-stimulus to 1,000 ms post-stimulus. EEG segments contaminated by amplifier clipping, bursts of electromyographic activity, or peak-to-peak deflection exceeding ± 100 μV were excluded from the average calculation. EEG segments were averaged separately for the target and standard stimuli. The EEG segments were averaged separately for target and standard stimuli. The number of average trials left after removal of the artifacts was 60 (target) and 240 (standard) for normal controls and 60 (target) and 240 (standard) for patients, respectively.The peak amplitudes and latencies for one ERP component, P3, were measured relative to the pre-stimulus baseline period (Figure 3). The positive peak between 300–500 ms was used to define the P3 components. To reliably observe the target effect, P3d was obtained by subtracting the ERPs in response to standard stimuli from those in response to target stimuli (Figure 4). The mean amplitudes of the P3d were measured between 300 and 500 ms.

P3 components were analyzed using repeated-measures analysis of variance (ANOVA), with Stimulus (target, standard) and Site (F_3_, F_4_, Fz, C_3_, C_4_, Cz, P_3_, P_4_, and Pz) as the within-subject factors and Group (CH, control) as the between-subjects factor.

For P3d components, ANOVA was conducted with Site (F_3_, F_4_, Fz, C_3_, C_4_, Cz, P_3_, P_4_, and Pz) as the within-subject factor and Group (CH, control) as the between-subjects factor. Degrees of freedom were corrected using the Greenhouse–Geisser epsilon.

## Results

### MoCa and MMSE scores

The mean MMSE scores did not differ between CH patients (28.32 ± 1.48) and control subjects (29.03 ± 0.57; *p* > 0.05); this also applied to the mean MoCa scores (patients, 27.71 ± 1.35; control subjects, 28.06 ± 1.63; *p* > 0.05).

### Behavioral data

There was no significant group difference in accuracy (control, 99.33%; patients, 96.65%; *F*(1, 30) < 1). There was also no significant difference in the appropriate reaction time between CH patients (460.32 ms) and controls (446.91 ms; *F*(1, 30) < 1).

### ERP data: P3 and P3d components

Across conditions, a significant main effect of Site for P3 was observed (*F*(2,60) = 12.70, *p* < 0.001, η2 = 0.297), indicating a centro-parietal scalp distribution with a maximum of 9.16 μV at Pz. Post-hoc tests revealed that, while there was significant difference between patients and controls (p = 0.034), the mean amplitude of P3 was larger for controls (7.28 μV) than patients (3.82 μV; p < 0.05, η2 = 0.141). No other effects reached significant level (ps > 0.1).

There was a significant main effect of Site for P3d was observed (*F*(2,60) =40.53, *p* < 0.001, η2 = 0.575), indicating a centro-parietal scalp distribution with a maximum of 11.00 μV for P3d at Pz. Post-hoc tests revealed that, while there was significant difference between patients and controls (p = 0.007), the mean amplitude of P3d was larger for controls (8.95 μV) than patients (5.78 μV; p < 0.05, η2 = 0.221). No other effects reached significant level (ps > 0.1).

The latencies of the P3 and P3d components did not show any significant effects (ps > 0.1).

Eight CH patients had pain on the left side of the head and seven patients on the right side. The amplitudes of P3 were similar between the pain and no pain sides (*F*(1,30) = 1.807, *p* > 0.05). This also applied to the P3d (*F*(1,30) = 0.057, *p* > 0.05).

## Discussion

The present study investigated possible impairments in the cognitive processing of CH, using target processing and a visual oddball paradigm. Compared with the control group, there was a decrease in the amplitude of P3/ P3d in the patients. Before, there were no experiments investigate the reduced P3 amplitude in the cluster headache patients during the cluster bout period but outside an attack. Many studies focused on the latencies of P3 during an attack. For example, Evers et al. reported that latencies for the endogenous ERP components were significantly increased during the cluster bout period compared with outside the cluster bout period. In healthy subjects [9,10], P3 latencies in particular were longer [17]. Positive findings regarding the amplitude of ERP components have been observed infrequently; the instances in which they were observed owed principally to a lower CH incidence and a reduced impairment in cognitive ability.

The P3 component will now be discussed. P3 is a generic name for a variety of relatively late, positive components with a centro-parietal or centro-frontal midline distribution [16,18]. Initially discovered in response to task-relevant, infrequent oddball stimuli [19] and found to be sensitive to the subjective probability assigned to the occurrence of the eliciting event [20], many cognitive mechanism models underlying this neural event have been proposed [21-24]. Notwithstanding these controversies, it is widely accepted that P3 latency reflects the length of stimulus evaluation processes, when a two-choice reaction time (RT) is required [25] and its amplitude is largely determined by stimulus relevance [26], the amount of attention allocated to the stimulus [27] and the task’s complexity [28].

There is a low abundance of early research pertaining to correlations of hypothalamus, cingulate cortex and frontal lobe activations with the thought, comprehension, memory and other cognitive function domains. However, Honda, et al. [29], who recorded auditory ERP using the standard oddball paradigm, reported a low-amplitude P300 in a patient with a hypothalamic lesion. May et al. [5] also reported that the right anterior cingulate cortex and hypothalamus were involved in the exchange process between pain and cognition. Knight et al. [30] compared electrophysiological indices of auditory selective attention between control subjects and patients with unilateral dorsolateral frontal lobe lesions; they observed that lesions in the frontal lobes reduced attention-related negativity and impaired behavioral performance. Accordingly, decreased P3/P3d amplitudes in CH patients are likely due to functional and structural changes in hypothalamus, frontal lobe and cingulate cortex.

Many functional neuroimaging studies have confirmed abnormalities in structures such as the hypothalamus, cingulate gyrus, prefrontal lobe, and insular lobe. For example, May et al. [7] also investigated CH patients using PET and reported that activation occurred in the ipsilateral posterior inferior hypothalamic gray, the contralateral ventroposterior thalamus, the anterior cingulate cortex, the ipsilateral basal ganglia, the right anterior frontal lobe, and both insulae during an acute CH attack triggered by nitroglycerin (NTG). Qiu et al. [6,31], who employed resting-state functional magnetic resonance imaging (RS-fMRI), reported altered regional homogeneity in the anterior cingulate cortex, posterior cingulate cortex, prefrontal cortex, and insular cortex; recently, they also confirmed the presence of abnormal brain functional connectivity of the hypothalamus. Morelli et al. [2] used fMRI to show that during typical pain attacks in CH patients, significant activation occurs in the hypothalamic region ipsilateral to the side in which pain is being experienced.

**Figure 5:**
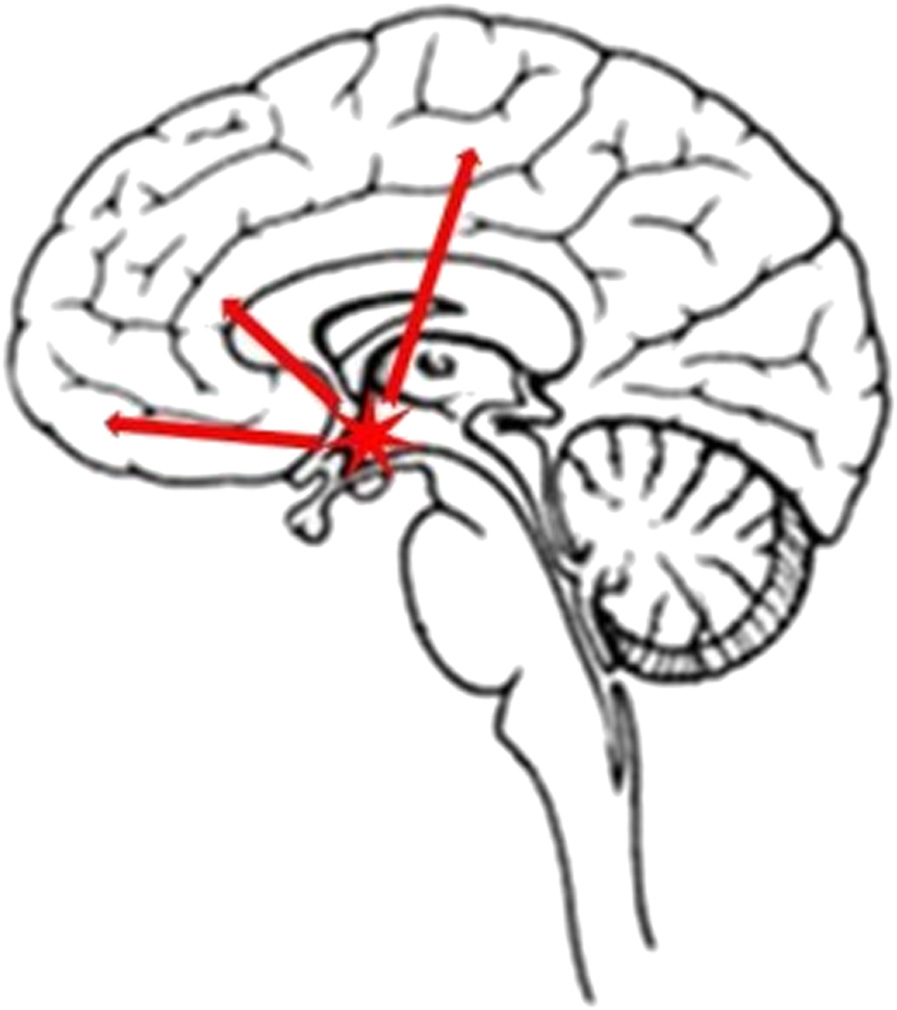
The hypothalamus communicated with the frontal lobe, parietal lobe, and cingulate gyrus in CH development.

The occurrence of abnormal functions in CH were due to the plasticity and hypersensitization of the cortics, this theory was well confirmed by many fundamental researches such as above mentioned [32]. Furthermore, the plasticity and hypersensitization of the cortics were also the reasons for the lack of habituation in electrophysiological examination of CH [33]. For instance, during the bout but not outside, cluster headache patients were chrarcterized by a pronounced lack of habituation of the brainstem blink reflex and a general sensitization of pain processing on the headache side. All of these could be due to the descending metabolism of the dopamine [34]. And the dopamine agonist, such as rotigotine had proven to be effective in treating chronic cluster headache [35]. Then in the related intracranial structure,hypothalamus as a part of a supraspinal network involved in the descending control of pain was payed close attention for all the time. The hypothalamus in cluster headache might be characterized not only by a neuronal dysfunction but even by changes in the membrane lipids [36]. Studies with the proton MR spectroscopy (1H-MRS) demonstrated that the NAA and the Cho/Cr metabolite ratio were reduced in the hypothalamus in CH patients when compared to healthy subjects [37,38]. Armando Perrotta, et al. [39] studied the functional activity of the diffuse noxious inhibitory control by evaluating the effect of the cold pressor test on the temporal summation threshold of the nociceptive withdrawal reflex, found that CH revealed a significant facilitation in temporal processing of pain stimuli during the active phase. So they hypothesized that there was a dysfunction of the supraspinal control of pain in CH, and possibly supported by an abnormal hypothalamic function. Furthermore, the P3/P3d amplitudes were dysfunctioning equally in both affected and not affected sides. These phenomena revealed that the influences on the cognitive processing of affected sides were same as the not affected. These phenomena supported that the cognitive processing of cluster headache patients was damaged whatever the headache on the right or left. The hypothalamus played the key role in above mentioned processes, not only involved in human cognitive processing, but also participate in the occurs of cluster headache.In accordance with the above discussion, we conclude that the hypothalamus is the central site involved in CH development, and that it communicates with other brain structures, such as the frontal lobe, parietal lobe, and cingulate gyrus (Figure 5). The metabolic disorders play the key role in these procedures. In ERP studies, these can manifest as disorders of the P3/P3d and many other components, and in neuroimaging studies, these can manifest as disorders in all the above mentioned regions. However, we have to admit that the evidences support the conclusion above-mentioned are poor, and our conclusion is in the preliminary stage. So in the future we need to conduct the further researches such as observing the patients with hypothalamic lesions by ERP.

In closing, we would like to restate the procedural decisions that can somewhat constrain interpretation of the present findings. First, we did not employ other neuropsychological indices in conjunction with ERPs. Second, CH patients out of bout were not included in our study. Third, if we had observed patients with hypothalamic lesions, then our results would have been more comprehensive; as such, there are many avenues via which future studies could expand upon our findings.

## Conclusion

Our results pertaining to P3/P3d provide evidence for dysfunction in the cognitive processing of CH patients. Furthermore, our findings emphasize the involvement of the hypothalamus in the pathophysiology of CH.

## Competing interests

The authors declare that they have no competing interests.

## Authors’ contributions

MD RW, ZD, XC, MZ and LR carried out the studies. And RW drafted the manuscript. MD RW participated in the design of the study and performed the statistical analysis. Professor JW participated in the design of the study. Professor SY, the PI of this study, conceived of the study and participated in its design and helped to draft the manuscript. All authors read and approved the final manuscript.
